# The importance of *Helicobacter pylori* eradication: a narrative review

**DOI:** 10.3389/fgstr.2026.1740221

**Published:** 2026-04-01

**Authors:** Orlaith Casey, Marta Dobric, Orlaith Kelly, Colm Antoine O’Morain

**Affiliations:** 1 Beacon Hospital Research Institute, Beacon Hospital, Dublin, Ireland; 2 Department of Gastroenterology, Connolly Hospital, Blanchardstown, Dublin, Ireland; 3 School of Postgraduate Studies, Royal College of Surgeons in Ireland, Dublin, Ireland; 4 School of Medicine, Trinity College Dublin, Dublin, Ireland

**Keywords:** epidemiology, eradication, gastric cancer, *Helicobacter pylori*, public health, screening

## Abstract

**Background:**

*Helicobacter pylori (H. pylori)* is a gram-negative bacterium infecting over 40% of the global population, with highest prevalence in low- and middle-income regions. Chronic infection leads to persistent gastritis and can result in peptic ulcer disease, dyspepsia and gastric adenocarcinoma. Despite its high pathogenic potential, population-based screening and eradication programmes remain limited, particularly in Europe.

**Methods:**

This narrative review summarisescurrent evidence on the epidemiology, clinical burden and eradication strategies for *H. pylori*. A literature search of PubMed, Embase and the Cochrane Database was performed to identify recent publications relating to *H. pylori* infection, consequences and screening approaches.

**Results:**

*H. pylori* has been classified by the International Agency for Research on Cancer (IARC) as a Group 1 carcinogen and is the leading cause of infection-attributable cancer worldwide, accounting for approximately 850,000 gastric cancer cases annually. Robust evidence demonstrates that eradication of *H. pylori* reduces the risk of peptic ulcer recurrence, dyspepsia, and gastric cancer incidence, with recent meta-analyses reporting a pooled relative risk reduction of up to 44%. Population-based eradication initiatives, such as the Matsu Islands study, have shown dramatic declines in *H. pylori* prevalence, peptic ulceration, and gastric cancer incidence. Current international guidelines, including the Maastricht VI/Florence and 2025 Taipei Global Consensus reports, now recommend universal eradication of confirmed infections and support population-level or family-based screening in high-risk regions. Ongoing European pilot studies, including the TOGAS initiative, aim to inform the implementation of gastric cancer screening programs. Concerns regarding antibiotic resistance remain, though recent evidence suggests that the risks are manageable with appropriate antibiotic stewardship.

**Conclusion:**

Population-based *H. pylori* screen-and-treat strategies represent a cost-effective and evidence-based approach to preventing gastric cancer. Further large-scale European studies are warranted to guide optimal implementation, timing, and cost-effectiveness of such programs.

## Graphical Abstract

**Figure:**
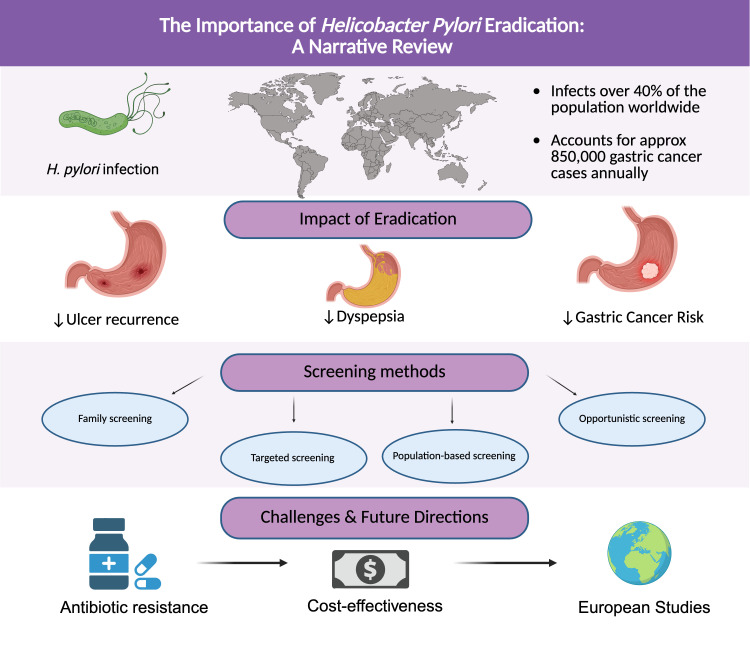
Created in BioRender. Casey, O. (2026) https://BioRender.com/6anpda1\.

## Introduction

*Helicobacter pylori (H. pylori)* is a gram-negative, flagellated, helical bacteria which is known to infect over 40% of the population worldwide, with massive geographical variations (1–4). It is believed that the bacterium is contracted in childhood, horizontally within families, and subsequently colonises the mucosa of the stomach and will persist there for many years (5). This is due to both its unique shape and its ability to produce urease which allows it to withstand the acidic environment of the stomach (6). It is also known to have distinctive abilities to evade the host’s immune response by adaptation of its surface molecules and modulation of effector T cell responses (7, 8).

The highest prevalence of *H. pylori* is seen in Africa (70%) and the lowest in Oceania (24%) (9). Higher prevalence rates are generally seen in countries with lower human development index (HDI) rankings, which may be explained by both poor antimicrobial stewardship and limited resources (3, 10).

The global prevalence of *H. pylori* is felt to be declining in adults, as shown in a meta-analysis published in 2024 which reported than the crude global prevalence has reduced from 52.6% before 1990 to 43.9% between 2015 and 2022. However, the same is not seen in childhood and adolescent prevalence estimates (3). In additional to this age-group discordance, there is also substantial heterogeneity between studies which limits the strength of causal and population-wide inferences that can be drawn from pooled estimates and suggests that global averages may obscure important regional and demographic differences.

Longstanding infection with the bacteria causes a state of chronic inflammation, which is often asymptomatic and can result in the development of precancerous lesions in the stomach such as atrophy, intestinal metaplasia and ultimately the development of gastric cancer (6, 8, 11).

H pylori was first discovered by Marshall and Warren in 1983, following successful culture of the bacteria. They first published a letter to the editor regarding their observation and followed this up with a full research article in 1984 (12, 13). They were subsequently honoured with the Nobel Prize in Physiology or Medicine in 2005 for their pioneering work.

An Irish clinical trial published in 1987 revealed that *H. pylori* status was a significant predictor of endoscopic relapse of duodenal ulcers. This was a follow-up study of patients with healed duodenal ulcers, who underwent repeat endoscopy at 12-months. Those who had recurrence of the bacteria were 66% more likely to have a relapse in ulceration, suggesting that *H. pylori* is a major causative factor in peptic ulcer disease. It also found that none of those who achieved successful eradication of the bacteria had histological gastritis (14). An Australian study published in 1988 confirmed these findings. They followed 100 patients with the infection and duodenal ulceration and found that in those who achieved eradication, 92% of ulcers healed, which was a statistically significant result. This is in contrast to those who did not achieve eradication, in whom 61% of ulcers healed and 84% had a relapse of ulceration (15).

In the 1990s, further studies were published which proved the link between the presence of the infection and an increased risk of development of gastric adenocarcinoma (16, 17). Parsonnet et al. published a study in 1991 which examined a cohort of 186 patients with gastric carcinoma compared to a similar size control group without gastric carcinoma. They found that 84% of those with gastric carcinoma had evidence of previous *H. pylori* infection, compared to 61% in the control group. They concluded from this study that *H. pylori* infection resulted in an increased risk of gastric carcinoma (16).

Given the established pathogenicity, global prevalence and carcinogenic potential of *H. pylori*, the rationale for eradication in the general population via gastric cancer screening programs is compelling. The following sections will explore in detail the benefits and challenges of *H. pylori* eradication.

## Methods

This narrative review article provides a comprehensive overview of the key findings in relation to the dangers of *H. pylori* infection and the importance of its eradication.

A comprehensive literature search was conducted to identify the most recent and relevant publications on *H. pylori* infection, its history, epidemiology, clinical consequences and screening methods. Databases searched included PubMed, Embase and the Cochrane Database, using free-text terms such as “Helicobacter pylori”, “Helicobacter pylori epidemiology”, “consequences of Helicobacter pylori eradication” and “gastric cancer”.

### The burden of *H. pylori*

*H. pylori* has been described as a class 1 carcinogen by the International Agency for Research on Cancer (IARC) and is the leading cause of infection-attributable cancer worldwide (9, 17–19).

It has been estimated to have 850,000 attributable cases of gastric cancer which is higher than those of Human Papillomavirus (HPV), which is described to have 730, 000 attributable cases and already has advanced screening programs in place in many countries worldwide. It is also believed to have more cases than Hepatitis B&C which have 550, 000 attributable cancer cases combined (9).

Non-cardia gastric cancer (NCGC) is estimated to be caused primarily by *H. pylori* in over 80% of cases and even this has been considered to be a possible underestimation, given the imperfections in data collection on *H. pylori* exposure (9). In addition to this, *H. pylori* has also been linked to cardia gastric cancer and mucosa-associated lymphoid tissue (MALT) lymphoma, which further increases its malignant burden (9).

### Clinical consequences

The clinical consequences of *H. pylori* infection are broad. Almost everyone chronically infected with the bacteria will develop gastritis due to the underlying chronic inflammation (13, 20). The most prominent consequences include peptic ulcer disease, dyspepsia and gastric cancer. Other consequences include mucosa associated lymphoid tissue (MALT) lymphoma, immune thrombocytopenic purpura (ITP) and iron deficiency anaemia (21–25).

The first global consensus on the management of gastritis was reached in Kyoto in 2015. Many clinical questions were addressed at this meeting including decisions around the clinical distinction of dyspepsia caused by *H. pylori* from functional dyspepsia and the treatment of *H. pylori* gastritis (20). The existing ICD-10 (International Classification of Diseases) classification for gastritis was felt to be outdated given that it did not account for the important aetiological factor of *H. pylori*. The proposed ICD-11 classification was an improvement. It also found that *H. pylori* gastritis should be defined as an infectious disease, irrespective of complications. 100% consensus was reached on the statement that *H. pylori* causes dyspepsia, while 94.7% consensus was reached on the statement that eradication of *H. pylori* is first-line treatment for *H. pylori-*infected dyspeptic patients.

With regard to the appropriateness of search and screen for *H. pylori* gastritis, unanimous consensus was reached on search and screen for *H. pylori* gastritis at an age before development of atrophic gastritis and intestinal metaplasia, depending on the epidemiological context.

Notably, the decision around all *H. pylori*-positive individuals receiving eradication therapy was found to have a high level of evidence, a strong grade of recommendation and 100% consensus (20).

Following on from this, the Maastricht Consensus Statement has evolved over many years since the first report published in 1997, which advised considering eradication for specific indications such as peptic ulcer disease (26). Initially, a ‘Test & Treat’ strategy was advised at the 2^nd^ Maastricht Consensus for those under the age of 45 presenting with persistent dyspepsia in those without gastro-oesophageal reflux disease (GORD) symptoms, use of non-steroidal anti-inflammatory drugs (NSAIDs) and alarm symptoms (27). The third report, published in 2007, advised that all those who have 1^st^ degree relatives with gastric cancer should receive eradication therapy. At this point, eradication of the infection was felt to have ‘potential’ in reducing the risk of gastric cancer (28). The ‘Screen & Treat’ strategy came to light in the Maastricht IV/Florence Consensus Report which was published in 2012. At this time, *H. pylori* eradication was felt to be a cost-effective method of gastric cancer prevention in high-risk populations (29). A stronger emphasis on population-based *H. pylori* screen-and-treat strategies was suggested in the 5^th^ Maastricht/Florence Consensus Report, following conclusions from the IARC working group meeting in December 2023 (30).

Most recently, the Maastricht VI/Florence Consensus report of 2022 noted that *H. pylori* infection is recognised as an infectious disease and included in the ICD-11 revision. It states that all infected patients should receive treatment, as opposed to only those with clinical evidence of infection. There was 100% agreement on the statement that *H. pylori* infection is the primary aetiological factor for gastric adenocarcinoma.

Additionally, in this most recent report, issues around the rising rates of antibiotic resistance are addressed, with emphasis placed on antibiotic stewardship when treating the infection. Again, ongoing suggestion for population-based screen-and-treat strategies were made (31).

**Figure 1:**
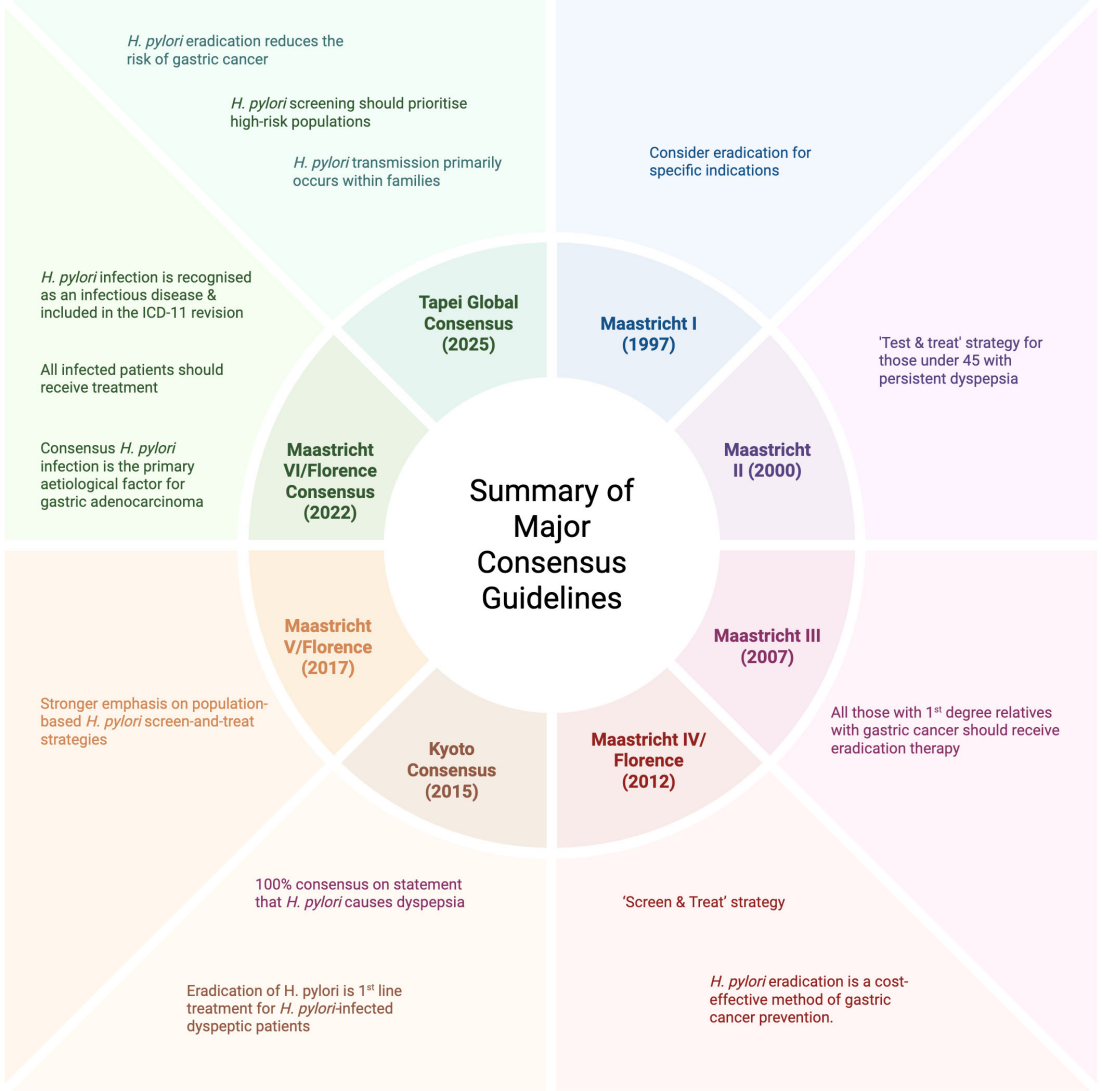
Summary of major consensus guidelines. Created in BioRender. Casey, O. (2026) https://BioRender.com/5ohh9hu.

In October of 2025, the Taipei Global Consensus published their second guideline with the most up-to-date consensus statements. It reported that *H. pylori* eradication reduces the risk of gastric cancer across all age-groups with the greatest risk reduction before the onset of premalignant conditions (32). It was also stated that *H. pylori* transmission primarily occurs within families, suggesting that family-based screening may be favourable in gastric cancer screening in the future. This consensus also agreed that *H. pylori* screening should prioritise high-risk populations with breath testing and SAT the preferred options (32). Ultimate conclusions from the Consensus guideline are that *H. pylori* eradication should be offered to all those with confirmed infection and that areas for further research and development include determining the optimal timing for screening as well as the long-term effects of screening (32). **Figure 1** provides a summary of the major consensus guidelines mentioned above.

#### Peptic ulcers

Following on from the above-mentioned pivotal publications in 1987 and 1988, a large and long-term population-based study from the Matsu Islands in Taiwan further corroborated the finding that *H. pylori* eradication resulted in prevention of peptic ulceration (14, 15, 33). This study, carried out between 2004 and 2018 on those aged 30 or older, analysed the outcomes of mass eradication of *H. pylori (*33). The entire population of the islands registered on the population registry were included. Understandably, prevalence rates dropped massively from 64.2% to 15%. Atrophic gastritis and intestinal metaplasia rates also fell, as did the incidence of gastric cancer, which was 9% in 2004, compared to 2% in 2018.

During this study, endoscopic examination of the stomach was also carried out. Upon commencement of the study in 2004, active peptic ulcers were seen in 11% of endoscopies while by 2018, following six rounds of mass screening and eradication, active peptic ulcers were seen in only 3% of endoscopies (33). Thus, it is reasonable to conclude that *H. pylori* eradication may both cure and prevent peptic ulceration (14, 15, 33).

#### Dyspepsia

The benefit of *H. pylori* eradication on symptoms of dyspepsia has been analysed in multiple randomised controlled trials (RCTs), which have found that dyspepsia is more likely to occur in control groups rather than those who have received *H. pylori* eradication (34, 35). A meta-analysis of these trials has produced a risk ratio of 0.82 (95% confidence interval 0.74-09.2) in favour of eradication (36). Although the number of included trials is small, there is low statistical heterogeneity which strengthens these findings.

Furthermore, a study from the UK, published in 2000, analysed those between the ages of 40–49 from 36 primary-care centres. 2324 participants of the 8455 who were eligible were found to be positive for *H. pylori* and were assigned to either treatment or placebo. 28% of the treatment group had dyspepsia or symptoms of gastro-oesophageal reflux compared to 33% in the placebo group, conferring a risk reduction of 5% (34).

#### Gastric cancer

Due to the ageing population, it is estimated that gastric cancer cases will increase from 968,000 in 2022 to 1.8 million by 2040. Consequentially, the estimated mortality from gastric cancer is set to increase from 660,000 in 2022 to 1.3 million by 2040 (37). Data reported in the IARC working group report suggest that the Western Pacific Region is expected to be worst affected in terms of new gastric cancer cases and deaths (9). It is Also expected that the number of new cases will be higher in countries with higher HDI (9).

The development of gastric cancer is well described by the gastric carcinogenesis cascade which was published in 1992 (38). Normal gastric mucosa, when disturbed by the presence of the bacteria and the possible addition of other risk factors such as a high-salt diet, will develop superficial gastritis. This progresses to chronic inflammation which can progress to atrophic gastritis. Consequentially, where there is a higher gastric pH as well as bacterial overgrowth and nitrate reduction, one can develop intestinal metaplasia and spasmolytic polypeptide-expressing metaplasia (SPEM). These precancerous lesions can develop into dysplasia, and in the context of ongoing chronic inflammation and reactive oxygen species, this can progress to carcinoma (38). This is a chronic and lengthy process which often takes decades.

Finally, the most harmful of *H. pylori*’s constellation of clinical consequences is that of gastric adenocarcinoma.

Evidently, eradication of the bacteria in an effort to prevent further cases of gastric cancer is encouraged. A meta-analysis published this year provides robust evidence for eradication therapy in gastric cancer prevention. In this meta-analysis, 11 RCTs and 13 observational studies were analysed. In the analysed RCTs, the pooled relative risk of gastric cancer following eradication therapy was 0.64 (95% confidence interval, 0.48-0.84). This is further corroborated in the observational study analysis with pooled relative risk of 0.56 for those who received eradication therapy (39). Thes findings demonstrate consistent relative risk reduction across randomised and observational studies. The IARC working group performed a systematic review of RCTs and deduced that the number needed to treat to prevent one case of gastric cancer was 228 (36).

It is important to note that there is somewhat limited data on *H. pylori* and gastric adenocarcinoma in Europe, perhaps due to lower prevalence rates. A new population-based cohort study published this year based on the Nordic population (Denmark, Finland, Iceland, Norway and Sweden), provides some of the missing data. A large cohort of 659,592 participants who received *H. pylori* eradication treatment, 1311 developed NCGC. This was compared to the entire Nordic population, and it was found that 11 years after treatment, the risk of development of NCGC was similar to that of the background population. This suggests that *H. pylori* eradication does indeed result in a decreasing incidence of NCGC (40).

Ultimately, *H. pylori* could be described as a childhood infection with geriatric consequences (5). The longer it is allowed to develop, the bigger the clinical consequences.

The adage “Better to build a fence at the top of the cliff than to park an ambulance at the bottom” rings true (41). This could be compared to the development of *H. pylori* screen-and-treat strategies in the prevention of gastric cancer.

### Gastric cancer screening

There are a variety of methods by which screening can be undertaken. These include targeted screening in specific high-risk populations, opportunistic screening, family screening and population-based screening (**Table 1**).

**Table 1:** Methods of screening.

1. Targeted screening: screening for specific high-risk populations e.g., geographic or ethnic groups
2. Opportunistic screening: screening on an *ad-hoc* basis during clinical interactions
3. Family screening: screening for those of family members who have been diagnosed with the bacteria
4. Population-based screening: widespread screening for the entire population

In the United States, the American gastroenterological association (AGA) recently published a clinical practice update on screening and surveillance in those at increased risk of gastric cancer. This clinical practice update is largely focused on the idea of targeted screening in identifiable high-risk groups, such as immigrants from areas with known high-incidence gastric cancer and those with a family history in a first-degree relative. One of the best practice advice statements from this update outlines that *H. pylori* should be screened for opportunistically in those believed to be at increased risk for gastric cancer. It also reports that those household contacts of a person testing positive for *H. pylori* should be tested (42). This publication is in contrast with the recent IARC working group report which concludes that there is now moderate evidence that population-based *H. pylori* screening will indeed reduce the incidence of gastric adenocarcinoma. It ultimately suggests that best-practice is population-based screening as it is quality assured and cost-effective (36).

### Screening methods

There are a variety of non-invasive methods for detection of *H. pylori* infection. They include 13C/14C Urea Breath Testing (UBT), stool antigen testing (SAT) and serology. The choice of non-invasive method is well-described in the IARC working group report (43). It is pertinent that the chosen test is locally validated within the population eligible for screening. This can be challenging, given the lack of data on the bacteria in certain regions. As such, the choice of test should be dependent on the “local disease context” (43). Further factors for consideration also include cost and availability of the desired test. More invasive screening methods such as endoscopy and biopsy are not recommended for use at a population-based level, mainly given that endoscopy is invasive and costly (43).

The targeted screening approach is one that is currently being considered in New Zealand, given the disproportionately high prevalence rates of *H. pylori* in Māori and Pacific populations (44). Studies have shown that *H. pylori* seropositivity in Pacific people is 3 times that of Europeans and in Māori people is it twice that of Europeans (45). The rates of peptic ulcer hospitalisation, gastric cancer incidence and mortality are also dramatically higher in those of Māori and Pacific ethnicity (44). Cost-effectiveness analyses have shown that a targeted screening approach for Māori people would result in an improved incremental cost-effectiveness ratio compared to that of the entire population (44).

A 2017 study conducted in Bhutan focused on a younger cohort of schoolchildren aged between 4 and 19 years old and involved serology testing for the bacteria (46). This study found that the prevalence as 66% overall with no statistically significant difference between gender or age subgroup. It did find that the prevalence of the bacteria was inversely correlated with the level of mother’s education and that households with fewer than 3 children had a lower prevalence than those with 3 or more children. This study highlighted possible options for targeted screening within this country, given that gastric cancer is the most common cause of cancer-related deaths in Bhutan.

A novel screening approach was studied in Changhua County in Taiwan which involved dual testing for *H. pylori* stool antigen (SAT) and faecal immunochemical testing (FIT). This study published in 2024 was a randomised clinical trial which utilised the existing Colorectal Cancer Screening platform to invite a cohort of 63, 508 participants for dual testing with SAT and FIT and 88, 995 for FIT alone. Participation rates were higher at 49.6% in the SAT and FIT group compared to 35.7% for FIT alone. Following adjustment for differences in screening participation, follow-up and patient characteristic, the invitation for both tests was associated with reduces rates of gastric cancer (47). This landmark study represents a practical method of gastric cancer screening implementation by combination with existing programs.

Gastric cancer prevention efforts are also underway in South America, with initiatives such as the HOPE-Hp-GC (Hospital and Outpatient Prevention Program to Eradicate *H. pylori* and Gastric Cancer) initiative which is based in Chile. This initiative involves screening with *H. pylori* pepsinogen tests and subsequent urea breath testing and treatment for those who are positive. Intermediate or high-risk participants based on *H. pylori* test and baseline comorbidities undergo gastroscopy with Sydney protocol biopsy and OLGA (operative link on gastritis assessment) evaluation (48). Further centres in Chile and Latin America are expected to join this initiative in 2025.

### Pilot studies - *TOGAS*

Despite having the second highest incidence of gastric cancer by WHO Region after the Western Pacific Region, no dedicated screening program exists for gastric cancer in the European Region (9). A number of pilot studies are currently underway within the European Union (EU) aimed at addressing the gaps in evidence required for the implementation of cancer screening programs. These include the “Strengthening the screening of Lung Cancer in Europe” or “SOLACE” study, the “PRostate cancer Awareness and Initiative for Screening in the European Union” or “PRAISE-U” study and the “TOwards Gastric cancer Screening Implementation in the European Union” Or “TOGAS” study. The TOGAS study includes a number of pilot studies and the consortium is composed of several representatives from a number of EU member states. The Pilot 1 consortium includes Slovenia, Ireland, Latvia, Poland, Croatia and Romania while the Pilot 2 consortium includes Germany, Spain, France, Lithuania, The Netherlands, Lativa, Portugal and Ireland.

TOGAS “Pilot 1” is focused on a screen-and-treat approach for individuals aged 30–34 using non-invasive serology for *H. pylori* detection and confirmatory UBT. This age group was selected in order to avoid intrafamilial transmission from parents to children and also to eradicate the bacteria prior to the development of precancerous lesions. Those individuals testing positive for the bacteria are prescribed quadruple therapy treatment and undergo a repeat UBT to confirm eradication.

The Pilot 2 study is based on an older population aged 50-74-years-old and is based on offering additional gastroscopy to those undergoing FIT-positive or surveillance colonoscopy. It aims to identify *H. pylori* prevalence, as well as the prevalence of gastric atrophy, intestinal metaplasia and gastric cancer in an asymptomatic cohort. This age-group was selected as it coincides with the onset of colorectal cancer screening in many European countries. The overall aim of the study is to “recommend the appropriate implementation of gastric cancer screening across the EU” (49).

### Downsides of screening

Some issues around screening have been raised previously including the possibility of increased rates of gastro-oesophageal reflux disease (GORD), increased risk of oesophageal cancer and increased use of antibiotic therapy. Additionally, there are many logistical and economic challenges which must be addressed.

#### Dyspepsia

Regarding reflux or dyspeptic symptoms in individuals having received *H. pylori* eradication, it was previously postulated that eradication could increase these symptoms. The idea behind this is that the presence of the bacteria can reduce acid production and its eradication can result in a rebound increase in acid secretion (50). A meta-analysis discussed in the IARC working group report evaluated the presence of reflux-symptoms in both a cohort of individuals having received *H. pylori* eradication and a control group (34, 51). Both studies found that there was a lower incidence of reflux symptoms in those having received eradication. It found a random risk ratio of 0.89 (95% confidence interval 0.77-1.04), highlighting that there appears not to be an association between eradication and reflux symptoms (36). However, the power of this inference is limited given the small number of studies.

#### Oesophageal adenocarcinoma

It is also believed that the presence of *H. pylori* infection is associated with a decreased incidence of oesophageal adenocarcinoma (52, 53). Logically, it may be believed that eradication of the bacteria would result in an increased risk of oesophageal adenocarcinoma. However, a recent large cohort study performed in the Nordic countries assessed the risk of oesophageal adenocarcinoma after *H. pylori* eradication. It included 661, 987 individuals who had been treated for *H. pylori* and compared this to the entire Nordic population. The standardised incidence ratio decreased over time following eradication, suggesting that there is no increased risk.

#### Antibiotic therapy

Screening for *H. pylori* would certainly involve a large increase in the use of antibiotic therapy for eradication. There are a number of reasons as to why *H. pylori* treatment is unsuccessful. It may be put down to the number of tablets to be taken daily, the duration of therapy, side effects of the therapy, high gastric acidity or bacterial load. However, the main reasons are felt to be due to antimicrobial resistance and compliance issues. Advice published in the IARC working group report suggests methods of improving antimicrobial stewardship. Local guidance for the use of antibiotics for *H. pylori* should always be followed in order to reduce the risk of antibiotic resistance (54). Eradication regimens should be carefully followed and retesting following treatment must be performed to ensure eradication has been achieved. Overall monitoring of eradication rates should be completed in order to manage the risk of antibiotic resistance. Clarithromycin resistance rates are rising, and it is currently though that >15% of *H. pylori* strains are resistant (54). Clarithromycin-resistant *H. pylori* has been declared as a high priority of antibiotic research and development by the World Health Organisation (WHO) (55). Additionally, there has been a noted increase in antibiotic resistance to metronidazole and levofloxacin. A systematic review and meta-analysis published in 2018 found resistance rates of clarithromycin, metronidazole and levofloxacin to be ≥15% in many WHO regions, with the exception of the Americas, South-East Asia and Europe (55). This raises concern for the ongoing use of older empirical regimens such as triple therapy, which is no longer recommended for empirical use in most guidelines (31). Quadruple therapy containing bismuth-salts in combination with tetracycline, metronidazole and a PPI is currently felt to be the most effective regimen (56). Bismuth possesses both synergistic effects with antibiotics and the ability to overcome metronidazole resistance, making it a highly effective medication in the treatment of *H. pylori (*57, 58).

Increased use of antimicrobial therapy has the potential to alter the gut microbiome, although this potential consequence can be improved by probiotic supplementation (59). However, chronic *H. pylori* infection itself is also known to affect the gut microbiota (59).

## Conclusion

In summary, a population-based screen-and-treat approach for *H. pylori* has the potential to contribute meaningfully to reductions in gastric cancer incidence over time. In order to achieve this, further robust European population-based studies are needed, in particular given the lack of data within Europe compared to Asian populations. Furthermore, comprehensive cost-benefit analyses are required to identify and implement the most cost-effective screening program in Ireland and across the EU.
